# Climate change and fire effects on a prairie–woodland ecotone: projecting species range shifts with a dynamic global vegetation model

**DOI:** 10.1002/ece3.877

**Published:** 2013-11-18

**Authors:** David A King, Dominique M Bachelet, Amy J Symstad

**Affiliations:** 1 Biological and Ecological Engineering, Oregon State UniversityCorvallis, Oregon, USA; 2 Conservation Biology InstituteCorvallis, Oregon, USA; 3 US Geological SurveyHot Springs, South Dakota, USA

**Keywords:** DGVM, drought, fire, MC1, niche-based species distribution models, *Pinus ponderosa*

## Abstract

Large shifts in species ranges have been predicted under future climate scenarios based primarily on niche-based species distribution models. However, the mechanisms that would cause such shifts are uncertain. Natural and anthropogenic fires have shaped the distributions of many plant species, but their effects have seldom been included in future projections of species ranges. Here, we examine how the combination of climate and fire influence historical and future distributions of the ponderosa pine–prairie ecotone at the edge of the Black Hills in South Dakota, USA, as simulated by MC1, a dynamic global vegetation model that includes the effects of fire, climate, and atmospheric CO_2_ concentration on vegetation dynamics. For this purpose, we parameterized MC1 for ponderosa pine in the Black Hills, designating the revised model as MC1-WCNP. Results show that fire frequency, as affected by humidity and temperature, is central to the simulation of historical prairies in the warmer lowlands versus woodlands in the cooler, moister highlands. Based on three downscaled general circulation model climate projections for the 21st century, we simulate greater frequencies of natural fire throughout the area due to substantial warming and, for two of the climate projections, lower relative humidity. However, established ponderosa pine forests are relatively fire resistant, and areas that were initially wooded remained so over the 21st century for most of our future climate x fire management scenarios. This result contrasts with projections for ponderosa pine based on climatic niches, which suggest that its suitable habitat in the Black Hills will be greatly diminished by the middle of the 21st century. We hypothesize that the differences between the future predictions from these two approaches are due in part to the inclusion of fire effects in MC1, and we highlight the importance of accounting for fire as managed by humans in assessing both historical species distributions and future climate change effects.

## Introduction

Land-use change and the spread of exotic species have caused profound changes to the Earth's biota (Whitney 1994; Vitousek et al. 1997). More recently, changes in plant phenology and distributions of species have been attributed to climate change (e.g., Menzel et al. 2006; Kelly and Goulden 2008). Although concurrent changes in land use and fire regimes are often confounding factors in such studies (Archer et al. 1995; Schwilk and Keeley 2012), the substantial warming expected over the next century is predicted to have large effects on landscapes and species distributions (e.g., Bakkenes et al. 2002; McKenney et al. 2007).

Climate change impacts may be particularly noticeable at ecotones with abrupt boundaries between contrasting vegetation types (e.g., Gosz 1992; Risser 1995). Here, modest shifts in climate may cause substantial shifts in the vegetation that will eventually occupy such sites. However, actual climate change effects on the biota are uncertain, given lags in species migration, the longevity of trees, and the legacy of land-use practices both today and over past millennia (Thuiller et al. 2008). Agriculture and urbanization have fragmented landscapes and altered fire frequencies, thereby affecting seed dispersal and the abundance of fire-dependent species (Nowacki and Abrams 2008).

Projected climate change effects on organisms are usually generated by one of two general approaches: (1) niche-based empirical climate envelope or habitat suitability models, or (2) process-based models. Empirical models commonly use statistical relationships with climate over the geographic range of a species to determine whether the projected future climate lies within the “environmental space” for that species (Dawson et al. 2011). Logistical advantages of this approach are that: (1) there are user-friendly software packages for conducting the analyses, and (2) the required future climate projection datasets (multidecadal means rather than time series) are readily available.

Process-based models typically simulate the dynamics of species or organismal classes as affected by demographic and/or physiological processes. Many process-based vegetation models have focused on forests and competitive interactions among tree species. For example, forest gap models (Shugart 1984; Bugmann 2001) simulate the diameter growth of individuals, as affected by average annual climate and shading from neighbors. Early results projected large shifts in forest composition in response to climate change, although with centuries-long time lags (e.g., Solomon 1986; Pastor and Post 1988). However, early gap models were often calibrated to yield zero growth at both the coldest and warmest extent of each species, thus ensuring that a species not be simulated outside of its observed temperature range (Bugmann 2001). In reality, many trees grow vigorously (with adequate water) when planted at the warmer limits of their natural distributions, implying an important role for biotic interactions in species range limits (Loehle 1998; Kramer et al. 2012).

More recently, dynamic global vegetation models (DGVMs) have increasingly been used to project future distributions of the vegetation that would occur in the absence of industrialized societies. Time series of climate projections from coarse-resolution general circulation models (GCMs) have been spatially downscaled to provide the required driving variables. DGVMs simulate ecosystem processes and transient dynamics of plant functional types or life-forms based on empirical biogeographic patterns and general physiological processes, including the effects of CO_2_ on production and water-use efficiency. The occurrence and effects of disturbance such as fire have only been included in a few DGVMs so far (e.g., Bachelet et al. 2003; Thonicke et al. 2010; Rogers et al. 2011). However, because climate change will likely alter fire frequencies and intensities, mechanistic representations of fire are needed to project future vegetation dynamics.

In a few cases, gap models have been incorporated in DGVMs to project the dynamics of particular tree species in competition with other plant functional types (Smith et al. 2001; Hickler et al. 2012), thereby allowing species-level projections based on transient mechanistic processes. Dynamic fire has seldom been included in such multiscale models (but see Keane et al. 2011).

In this paper, we examine how the combination of climate and fire influence historical and future distributions of ponderosa pine, as simulated by a DGVM, along a grass–tree ecotone in the Black Hills of South Dakota, USA. We then compare our simulations to recent empirical projections of future suitable habitat for ponderosa pine in the Black Hills. We hypothesize that the substantial differences in the projections from these two approaches are due in part to the inclusion of fire effects in our model, and we highlight the importance of the influence of fire on both historical species distributions and the effect of future climate change.

We used the MC1 DGVM (Bachelet et al. 2001; King et al. 2013) to assess climate change effects at Wind Cave National Park (WCNP), South Dakota, USA, which lies near the eastern range limit of ponderosa pine (*Pinus ponderosa*). Rather than species, MC1 models life-forms and vegetation type distributions, as well as the associated dynamics in carbon stocks and fluxes. However, because ponderosa pine comprises nearly all of the tree biomass of the park and the Black Hills in general (Cogan et al. 1999; DeBlander 2002), we calibrated the model for this species, taking advantage of the fact that MC1 simulates fire, which is known to influence the distribution and structure of ponderosa pine forests (Brown and Sieg 1999; Allen et al. 2002). We refer to the revised model as MC1-WCNP. Thus, our assessment is essentially a projection of the competitive interactions between ponderosa pines and grassland, as mediated by fire. Once a satisfactory simulation of the current vegetation in the park was obtained, we projected the change in vegetation over the 21st century under three downscaled GCM climate trajectories, chosen to represent the range in climate change projections included in the 4th assessment report of the IPCC (IPCC 2007).

## Materials and Methods

Our general approach is as follows:

1. Alter the MC1 model to simulate ponderosa pine in the Black Hills and the observed abrupt spatial transition from forest to grassland.
2. Adjust the fire ignition thresholds of the model to approximate the observed pine–grassland ecotone by the end of the spinup and historical simulations, during which dynamic fire affects the balance between trees and grass.
3. Simulate vegetation dynamics throughout the 21st century with spatially downscaled climate inputs from three selected GCMs for three management scenarios: natural fire, fire suppression (with severe fires allowed to escape), and regularly prescribed fires (approximating current management practices at WCNP).

Our mechanistic approach contrasts with that taken with niche-based species distribution models, which commonly (1) use correlations to determine the “suitable habitat” of a species from presence–absence data and (2) assess changes in the spatial distribution of the suitable habitat for future climate scenarios, assuming the historical correlations will remain valid in the future. The niche-based approach does not consider transient dynamics of the vegetation nor the evolving land management practices that our model does consider.

### Site

Wind Cave National Park is located on the southeastern edge of the Black Hills in South Dakota, at the boundary between ponderosa pine forests in the northwest highlands of the park and mixed grass prairie on the lower, flatter southeast foothills. WCNP encompasses 11,400 ha of natural vegetation (a 2012 addition to the park is not considered here) and is located at 43.5**°**N lat., 103.5**°**W lon., with elevations varying between 1100 m and 1530 m. Mean annual precipitation is approximately 48 cm yr^−1^, half of which falls from May through July. WCNP is managed to maintain the native flora and fauna, including bison, elk, pronghorn antelope, and prairie dogs, and the extensive cave system for which it is named. Management includes population control of native grazers and historical fire suppression followed by a prescribed fire program that began in the 1970s.

**Figure 1:**
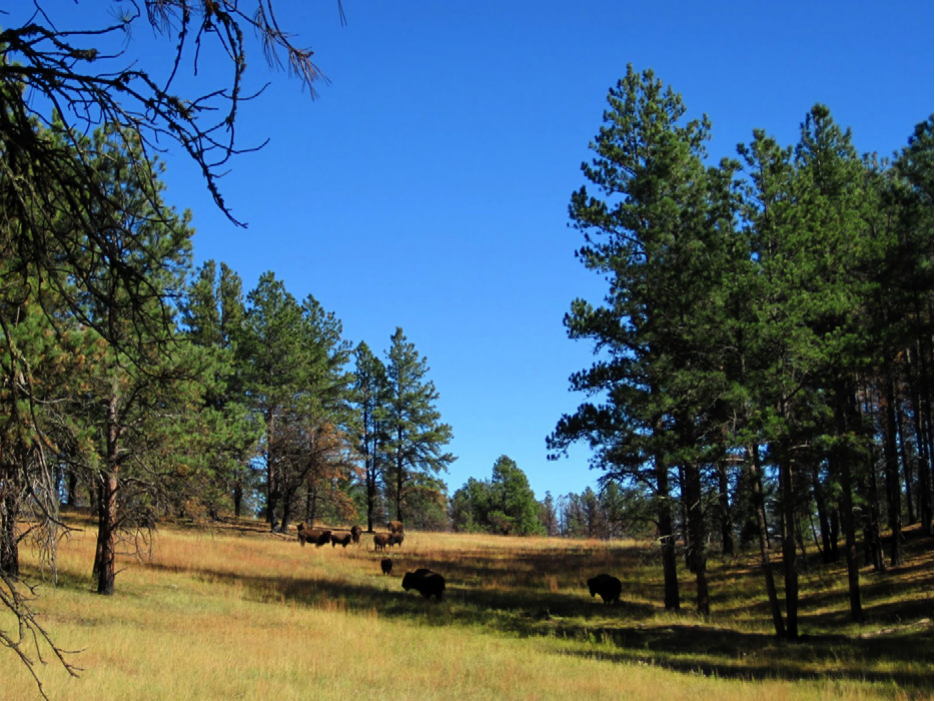
Typical occurrence of pure stands of ponderosa pine at Wind Cave National Park.

Two major vegetation types occur at WCNP: mixed grass prairie and ponderosa pine forest (Cogan et al. 1999). The forest comprises nearly 30% of the park, occurring primarily at higher elevations. Deciduous broadleaf trees are rare, and ponderosa pine comprises nearly all of the carbon attributable to the evergreen needleleaf life-form simulated by the model. The typical monospecific dominance by ponderosa pine is illustrated in Fig. 1.

### Data inputs

Required inputs to the MC1 model are soil depth, texture, and bulk density; annual average atmospheric CO_2_ concentration; and monthly average climate variables (monthly precipitation, mean vapor pressure, and means of daily maximum and minimum temperatures). Historical climate data (1895–2008) were acquired from the PRISM group (Daly et al. 2008) at 30-arc-second resolution (∼670 m EW x ∼930 m NS at the latitude of WCNP). Soils data from Kern (1995, 2000) were downscaled to the same resolution as the climate data.

Twenty-first century climate projections were acquired for three GCMs that span much of the range in temperature increases associated with the IPCC SRES A2 greenhouse gas emission scenario (Nakićenović et al. 2000): CSIRO Mk3 (Gordon 2002), Hadley CM3 (Johns et al. 2003), and MIROC 3.2 medres (Hasumi and Emori 2004) (henceforth CSIRO, Hadley, and MIROC). GCM future projections were downscaled using the delta or anomaly method of Fowler et al. (2007). For each climate variable and each future month, anomalies between future and mean monthly historical (1971–2000) GCM-simulated values were calculated for each GCM grid cell over the conterminous US. We used differences for temperature and ratios for precipitation and vapor pressure (capped at a maximum value of five). Anomalies were then downscaled to the 30-arc-second grid using bilinear interpolation and applied to the monthly historical PRISM baseline (1971–2000) available at the same spatial scale. The resulting future mean temperatures and precipitation were close to the PRISM means for 2001–2008 at WCNP (King et al. 2013). For the Upper Missouri River Basin (which includes WCNP) in the 2040s, the CSIRO climate is wetter and slightly cooler than the ensemble mean of the GCMs considered here, whereas MIROC yields one of the hottest and driest climates (Daniels et al. 2012; Fig. 5). (No comparison was available for Hadley.)

### Model description

MC1 is a process-based dynamic global vegetation model (DGVM) that simulates vegetation distribution, biogeochemical cycling and wildfire, and their interactions. The model simulates competition between trees and grasses, where the latter term refers to all nonwoody life-forms, including forbs and sedges, but does not simulate individual species. MC1 simulates the sizes of all plant and soil carbon and nitrogen pools and the fluxes between them, as well as hydrological fluxes. This linkage of pools and fluxes is accomplished with process-based equations, as is typical for DGVMs. Comprehensive documentation of MC1 and its mode of operation are given by Bachelet et al. (2001).

**Figure A1:**
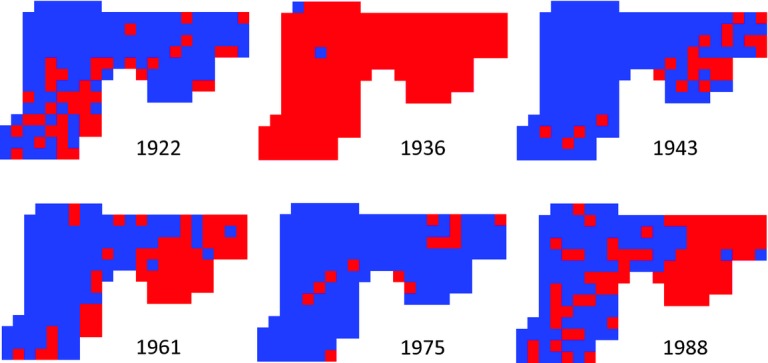
Representative patterns of simulated natural fire by the MC1-WCNP DGVM for Wind Cave National Park, South Dakota USA, including 1936, the year of greatest simulated fire extent. Red indicates burned cells.

MC1 has commonly been run at resolutions of 30-arc seconds to 0.5° (Bachelet et al. 2003; Lenihan et al. 2003; Rogers et al. 2011). Each grid cell is simulated independently, without cell-to-cell communication. However, drought conditions that trigger fires are often region-wide, resulting in similar fire effects across contiguous cells. For the WCNP historical run, this approach simulates fire across contiguous blocks of cells for the more severe fire years, as shown in Appendix 1, Fig A1. The model was formulated to simulate the potential vegetation that would occur without direct intervention by industrialized societies. However, applications of MC1 have included effects of humans on vegetation through cattle grazing and fire suppression as well as direct (CO_2_) and indirect (climate) effects of increasing greenhouse gas concentrations (Bachelet et al. 2000; Rogers et al. 2011).

MC1 consists of three linked modules that simulate (1) plant biogeography, (2) biogeochemistry (i.e., carbon, nitrogen, and water fluxes and pools), and (3) fire occurrence, behavior, and effects. The model is run in four sequential phases: equilibrium, spinup, historical, and future. During the equilibrium phase, the vegetation type is initialized, and the carbon pools equilibrated for fixed fire return intervals that depend on vegetation type, using averaged monthly climate inputs. During the spinup phase, the model is run iteratively with about 100 years of detrended historical climate data to allow for readjustments of vegetation type and carbon pools in response to dynamic wildfires. During the historical phase, the model is run with transient historical climate data starting in 1895. During the future phase that follows, the model uses downscaled climate data derived from GCM anomalies as described earlier.

#### Biogeography module

The biogeography module simulates vegetation types and lifeform mixes. This module projects changes in vegetation type through time, depending on temperature- and precipitation-based thresholds, and on biomass thresholds that are compared with carbon pools produced by the biogeochemical module. Life-forms include four types of trees (evergreen and deciduous needleleaf, evergreen and deciduous broadleaf) and two types of grasses (C3 and C4). Both tree and grass life-forms are always projected to coexist, although their relative abundance depends on climatic effects on growth (simulated by the biogeochemistry module) and fire effects (simulated by the fire module). Lifeform mixtures and the sizes of tree and grass carbon pools determine the potential vegetation type from among 38 possibilities, 14 within the temperate zone.

In transient mode (spinup, historical, and future), the mixture of tree types (evergreen and deciduous needleleaf, evergreen and deciduous broadleaf) is determined every year as a function of minimum temperature of the coldest month and growing season precipitation. For this purpose, climate inputs are smoothed to better represent the inertia of vegetation to short-term climate variability (Daly et al. 2000). Pure tree types are simulated for climate regimes defined by thresholds of warm season precipitation and mean minimum temperature of the coldest month. Gradations from one tree type to another occur along the existing climate gradients. Thus, shifts in tree type (e.g., evergreen needleleaf vs. deciduous broadleaf) are determined in a fashion analogous to that of niche-based models, but the plant biomasses that define the tree–grass ecotone are determined mechanistically by the biogeochemistry and fire modules.

#### Biogeochemistry module

The biogeochemistry model is a modified version of the CENTURY model (Parton et al. 1987; Metherell et al. 1993), which simulates the cycling of carbon and nitrogen among ecosystem components, including plant parts and multiple classes of litter and soil organic matter. This module also simulates actual and potential evapotranspiration (AET and PET) based on the Penman equation and soil water content in multiple soil layers. Tree and grass production rates are based on maximum monthly rates that are interpolated from lifeform-dependent parameter values, depending on the mixture of tree and grass life-forms set by the biogeography module. Maximum production rates are multiplied by temperature-, water-, and atmospheric CO_2_-related scalars that differ between trees and grasses (Bachelet et al. 2001). The water scalars are based on the ratio of available water (monthly precipitation plus plant available soil moisture) to monthly PET. Because PET is influenced by temperature, the model includes interactions between temperature and available water. In the case of trees, an additional scalar related to leaf area index (LAI, defined as one-sided leaf area per unit ground area) approximates the fraction of incoming light intercepted by trees. In the case of grasses, scalars incorporating the effects of shading by trees and standing dead grass are also included. A CO_2_ enhancement effect increases water-use efficiency and productivity as a logarithmic function of atmospheric CO_2_ concentration.

#### Fire module

The fire module simulates the occurrence, behavior, and effects of fire and was designed to project large, severe fires that account for the bulk of observed fire impacts in the conterminous United States (Lenihan et al. 1998, 2008). The module includes a set of mechanistic fire behavior and effects functions (Rothermel 1972; Peterson and Ryan 1986; Van Wagner 1993) embedded in a structure that enables two-way interactions with the biogeochemistry and biogeography modules. Live and dead fuel loads in 1-h, 10-h, 100-h, and 1000-h fuel classes (i.e., quick drying fine fuels to slow-drying coarse woody debris) are estimated from the carbon pool sizes produced by the biogeochemistry module. Allometric functions are used to calculate total height, crown base height, and bark thickness for an average-sized tree as a function of carbon pool sizes. The fractions of tree carbon pools killed by fire are functions of simulated fire intensity, crown position, and bark thickness, with complete mortality occurring in the case of crown fires.

The moisture contents of the fuel classes and the potential fire behavior are calculated each day using pseudo-daily data generated from simple interpolations of monthly climate inputs between mid-month values (Lenihan et al. 1998, 2008). Potential fire behavior (including rate of spread) is calculated each day based on daily-interpolated fuel loads, their moisture contents, and weather. Potential fire behavior is modulated by vegetation type, which affects fuel properties and realized wind speeds (higher for grassland than forest). Actual fire is simulated whenever the calculated rate of spread is greater than zero, and user-specified thresholds are exceeded for the fine fuel moisture code (FFMC) and the build-up index (BUI) of the Canadian fire weather index system. These two indices are inverse functions of fine fuel and coarser fuel moisture contents, respectively, as specified by Van Wagner and Pickett (1985). During each year for which fire is simulated for a given cell, only one fire is simulated on the first day when all thresholds are exceeded. As the daily inputs to the fire module are generated in a deterministic fashion, there is no stochastic variation in fire behavior or other outputs among multiple runs with the same inputs and initial conditions.

### Calibration of MC1-WCNP for WCNP

To apply our general lifeform-based model to ponderosa pine in the southern Black Hills, we adjusted the parameters and functions affecting tree geometry, drought effects, tree productivity, and fire dynamics, as described in Appendices 2 (including Table A2) and 3. We halved MC1's maximum LAI asymptote, based on data of Cannell (1982) and reduced the sensitivity of tree production to water deficits to be appropriate for ponderosa pine, a drought tolerant but shade-intolerant species with less dense foliage than more shade-tolerant species (Oliver and Ryker 1990; Niinemets and Valladares 2006). We adjusted the maximum net primary production for trees to yield an aboveground live tree carbon mass of 7500–9000 g C m^−2^ for old unburned forests at WCNP, near the upper end of the range inferred from plot measurements within the park (A. Symstad & D. Swanson, unpubl. data).

Before the advent of fire suppression, ponderosa pine forests were subject to less severe fire effects than those typically simulated by MC1 (Allen et al. 2002), and surface fires were common in the Black Hills in presettlement times (Brown and Sieg 1999). Hence, we altered the fire module such that simulated crown fires are rare in the more open mature forests that developed under presettlement fire frequencies in this area. Other changes to better simulate the grass–tree ecotone at WCNP are described in Appendix 2. In addition, we decreased the CO_2_ effect downward from a 25% to a 15% increase in production with doubled CO_2_, while maintaining the corresponding 25% decrease in transpiration, based on recent long-term studies of *in situ* CO_2_ impacts on vegetation (Appendix 2). This CO_2_-driven decrease in the transpiration rate slows the drawdown in soil moisture and increases tree resilience during drought periods.

Rogers et al. (2011) added an algorithm to MC1 to simulate intentional fire suppression by humans using thresholds for three fire intensity metrics: rate of spread (ROS), fireline intensity (FLI) and energy release component (ERC). They chose thresholds for the Pacific Northwest region where dense forests have high fuel loads and set them at 100 ft min^−1^ (0.51 m s^−1^) for ROS, 900 Btu ft^−1^ s^−1^ (3.1 MW m^−1^) for FLI, and 60 Btu ft^−2^ s^−1^ (0.68 MW m^−2^) for ERC. We found that, using these values, no fires could occur during the historical period at WCNP (which is inconsistent with actual fire history) and practically none under future conditions. In addition, our changes in fuel partitioning resulted in lower fine fuel loads than the version of MC1 used by Rogers et al. (2011), further reducing fire intensity. We thus reduced the three thresholds to 45% of their original values, allowing one fire on most of the forested cells at WCNP during an extremely hot and dry period in the 1930s.

### Simulation protocol

We ran MC1-WCNP using combinations of future climate and fire management scenarios (Table 1). Fire scenarios were determined in consultation with WCNP fire management specialists to reasonably represent their operations. During the equilibrium phase, the model was run iteratively for up to 3000 years with monthly climate means for 1895–1950 to equilibrate the most resistant soil carbon pool. The spinup phase was run iteratively with a detrended 1895–2008 time series for a total of 1140 years to produce a quasi-equilibrium state in net biome production, as affected by weather- and vegetation-dependent fires. Historical simulations began in 1895, and future simulations were run from 2001 (first GCM anomaly) to 2100. We also ran simulations without fire for both spinup and historical phases to illustrate its role in determining the grass–tree ecotone.

**Table 1:** Future fire management scenarios simulated by MC1-WCNP for Wind Cave National Park. Future climates downscaled from three general circulation models, CSIRO Mk3, Hadley CM3 and MIROC 3.2 medres (IPCC 2007), for the A2 anthropogenic emission scenario (Nakićenović et al. 2000).

Scenario	Description
Natural fire	Default fire simulation by MC1, with no more than one fire ignition per year at the time that fire ignition thresholds are first exceeded. Natural fire is simulated for spinup and historical phases and for each of the three future climate scenarios.
Fire suppression	Suppression of all potential natural fires, except those exceeding fire severity thresholds (Materials and Methods: *Calibration for WCNP*). Natural fire simulated through 1940, followed by fire suppression for the rest of the historical period and for each future climate scenario.
Prescribed Fire	Fire set on September 15 at 11 year intervals for each future climate scenario starting in 2001, each fire causing 20% tree mortality. No natural fires.

Spinup and historical simulations were run with grazing implemented (30% of monthly aboveground grass NPP removed, April through September). Threshold values for fine fuel moisture code (FFMC) and build-up index (BUI) were set at 90.4 and 80, respectively. These values result in the projection of one to three fires over the historical period for most cells in the wooded northwestern part of the park and 6–15 historical fires per cell over the somewhat warmer and drier easternmost part of the park. These frequencies are within the presettlement fire frequency range reported by Leenhouts (1998) for the Black Hills and adjacent grasslands.

The evergreen needleleaf tree type was projected to dominate throughout the spinup and historical runs and for two of the three future climates. However, for the wetter CSIRO climate, a large deciduous broadleaf component was at times projected. Such a rapid change seemed unreasonable because the current dominants must die if they are to be replaced by other species of similar or shorter stature (such as deciduous trees in the Black Hills), and there must be seed sources for the invaders. Given the rarity of deciduous trees in the park (Fig. 1; Cogan et al. 1999) and the general observation that existing trees grow faster in wet years than dry years (favoring the continued dominance of ponderosa pine), a substantial deciduous tree invasion seems quite unlikely during the 21st century under the CSIRO climate. In addition, current regeneration of deciduous trees is impeded by browsing ungulates at WCNP (Ripple and Beschta 2007). We therefore constrained the tree type to evergreen needleleaf for our 100-year future projections.

## Results

### Historical projection

**Figure 2:**
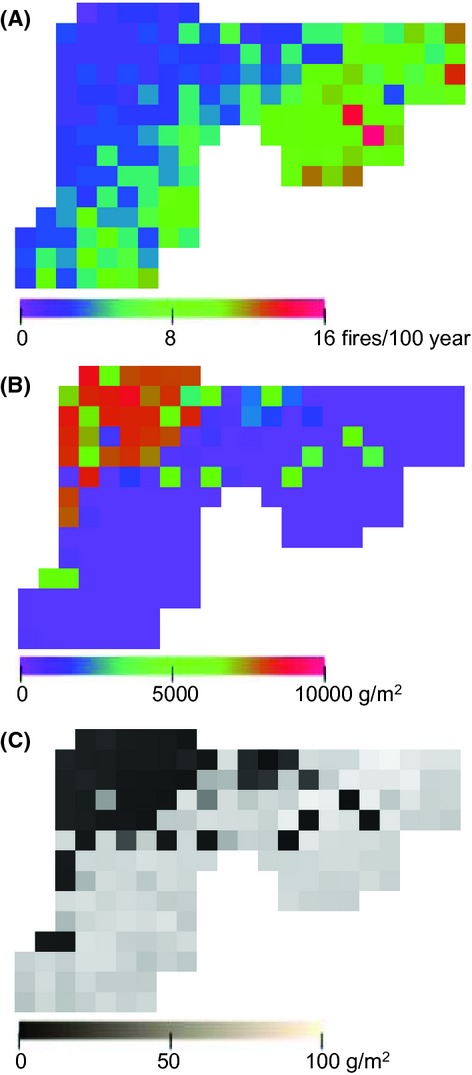
Simulated (A) natural fire frequency, and associated annual maximum (B) live aboveground tree C and (C) live aboveground grass C by the MC1-WCNP dynamic global vegetation model for Wind Cave National Park, SD using PRISM historical climate data. Fire frequencies are for 1901–2000; vegetation C masses are for the year 2000. An equiangular projection is used in this and subsequent figures of the park. Each grid cell is 30 arc seconds on all sides (∼670 m EW x ∼930 m NS at this 43.5° latitude).

The calibrated model simulates the observed proportions of the major vegetation types at WCNP (Table 2) with evergreen needleleaf forest in the northwest section of the park (Fig. 2B). Simulated fire frequencies are lower at these higher elevations (Fig. 2A, Appendix 1, Fig. A1), which are cooler and receive more precipitation than the southern and eastern parts of the park. Although the model does not capture the fine-scale patchiness in the distribution of trees over the entire landscape, it does simulate the existing distinct boundaries between wooded and grassland areas.

**Table 2:** Observed versus simulated percentages of land occupied by general plant groups at Wind Cave National Park. Observed values based on total areas of plant communities mapped by Cogan et al. (1999) over the park. MC1 simulated values for year 2000. Vegetation categories are combinations of the vegetation types from MC1 that correspond to the general classes of Cogan et al. (1999). For both MC1 and MC1-WCNP, the forest to woodland transition occurs as live tree C declines below 3000 g C m^−2^, the woodland – shrubland transition occurs at live tree *C* = 1150 g C m^−2^ and the shrubland – grassland transition at live tree *C* = 80 g C m^−2^.

Vegetation category	Observed %	Simulated %
Evergreen needleleaf forest or woodland	28.6	27.2
Deciduous broadleaf forest or woodland	0.3	0
Evergreen needleleaf shrubland	0	5.0
Deciduous broadleaf shrubland	7.5	0.0
Grassland	61.9	67.8
Other	1.7	0

**Figure 3:**
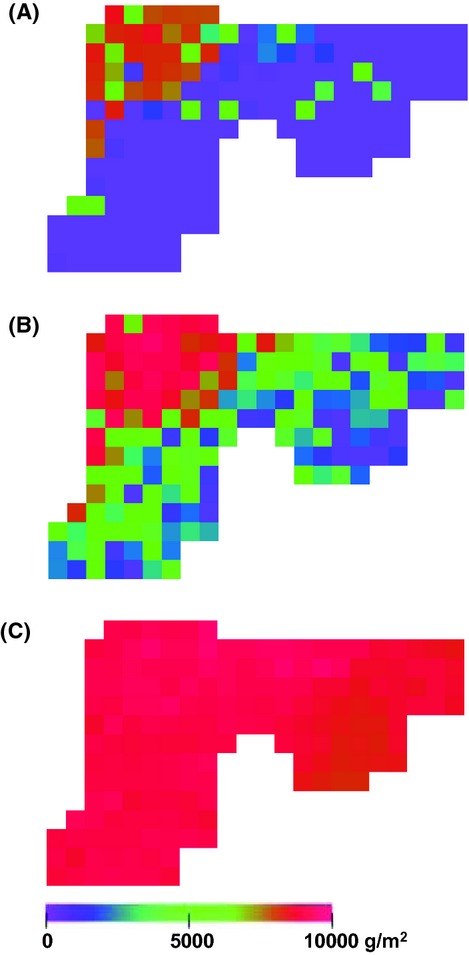
Simulated live aboveground tree C for year 2000 by the MC1-WCNP DGVM for Wind Cave National Park for (A) natural fire, (B) fire suppression, and (C) no fire during both the spinup and historical simulations in all cases.

**Figure 4:**
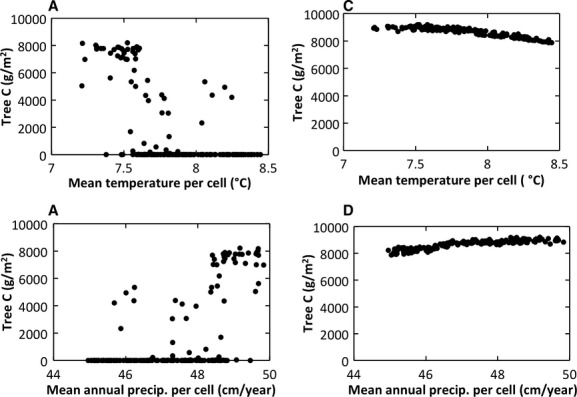
Mean aboveground live tree C per grid cell with fire (A and B), and without fire (C and D), as simulated by the MC1-WCNP DGVM for 1895–2000 for Wind Cave National Park using PRISM historical climate at 30 arc seconds.

Tree distribution is strongly dependent on simulated fire effects (Fig. 3), particularly during the 1140-year spinup phase, when a dynamic fire regime is established and the associated C pool sizes determine the initial conditions of the historical phase. Trees occupy most of the park area with fire suppression (allowing the escape of severe fires; Fig. 3B), but only in the absence of fire does stable forest cover the entire park (Fig. 3C). With natural fire, substantial live tree biomass is simulated for nearly all cells with mean annual temperature (Tmean) <7.6**°**C or mean annual precipitation (PPTmean) >48.5 cm (Fig. 4A,B). This pattern is greatly altered when fire is turned off during spinup and historical phases (Fig. 4C,D). With fire turned off, mean live tree biomass per cell is negatively correlated with mean annual temperature (*r* = -0.89, *P* < 0.001) and positively correlated with mean annual precipitation (*r* = 0.87, *P* < 0.001).

### Future climates

**Figure 5:**
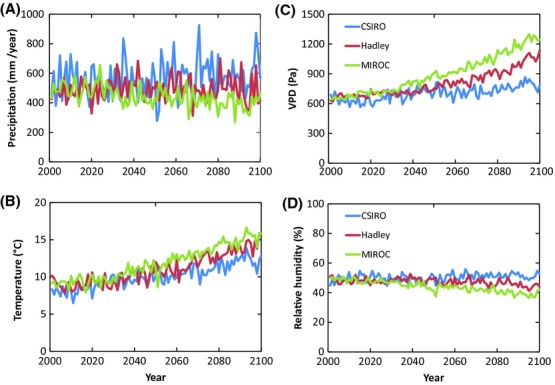
Projected future annual (A) precipitation, (B) mean temperature, (C) mean vapor pressure deficit and (D) mean relative humidity for the Wind Cave park headquarters cell, as downscaled from three general circulation models, CSIRO Mk3, Hadley CM3 and MIROC 3.2 medres (IPCC 2007), under the A2 emission scenario (Nakićenović et al. 2000).

All three downscaled climate scenarios show marked increases in temperature but diverge in precipitation projections for WCNP (Fig. 5). CSIRO projections are wetter and warmer than the present. Hadley temperatures increase substantially (>5^°^C by year 2100), but annual precipitation remains similar to present conditions. MIROC projections are the hottest and driest of the three. These differences become increasingly evident in the second half of the 21st century when the climates diverge. Vapor pressure deficit increases for all three scenarios but more so for Hadley and MIROC because of their large increases in temperature and modest declines in relative humidity (Fig. 5).

### Future fire occurrence

**Figure 6:**
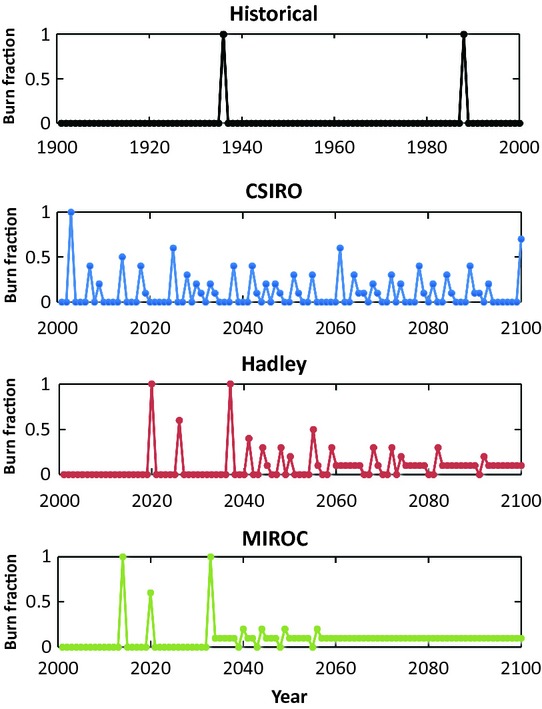
Fire ignitions (nonzero points) and grid cell fraction burned (magnitude of each point) simulated by MC1-WCNP for a representative forested cell (midrange in number of historical fires per forested cell) for the historical climate and downscaled future climates from three general circulation models; CSIRO Mk3, Hadley CM3 and MIROC 3.2 medres (IPCC 2007), under the A2 emission scenario (Nakićenović et al. 2000). For a forest grid cell, the fraction burned is less than 1 for fires that occur less than 10 years since the last fire; it has a value of 0.1 if a fire occurred in the preceding year.

The simulated ignition frequency of natural fires for all three future climates is much greater than during the historical period (Fig. 6). The effective burn frequency, defined as the sum of the fraction of the cell burned over a given time period, is 2.0, 10.7, 8.9, and 9.3 fires per century for the historical period, the CSIRO, Hadley, and MIROC 21st century projections, respectively, for a representative forested cell (Fig. 6). This burn frequency for wooded areas is lower than the associated ignition frequency of 39, 44, and 65 ignitions per century for CSIRO, Hadley, and MIROC, respectively, because we calibrated the model to reduce the burn fraction as fire return interval declined below 10 years, the time required for the fine fuels to increase to prefire levels (Appendix 2).

**Figure 7:**
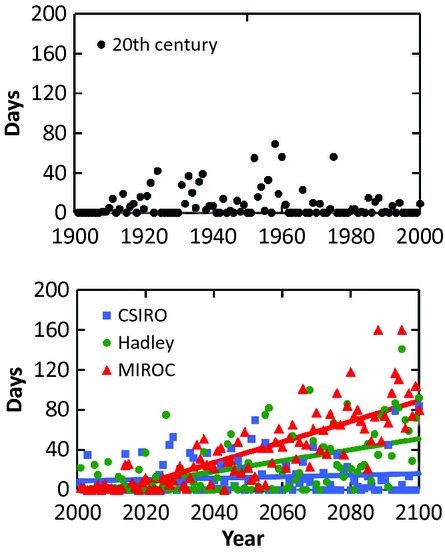
Number of days per year that the build-up index (BUI) exceeds its fire ignition threshold as simulated by MC1-WCNP for a representative forested cell (midrange in number of historical fires per forested cell) for the 20th century and three future climate projections from the general circulation models, CSIRO Mk3, Hadley CM3 and MIROC 3.2 medres (IPCC 2007), under the A2 emission scenario (Nakićenović et al. 2000). The colored lines are linear regression fits to the data for the correspondingly colored points.

The projected increase in fire ignition frequency is associated with an increase in the fire danger indices, as shown for a representative forested cell in Fig. 7 for BUI, which reflects longer-term drying of larger fuels. During the 20th century, the model predicts that BUI exceeds the fire ignition threshold for an average of 8.5 days per year, with a maximum value of 69 days for 1 year during the drought of the 1950s. The number of high BUI days increases substantially over the 21st century for Hadley and MIROC. However, the difference in fire danger among future climate scenarios only becomes apparent after 2040, when climate scenarios clearly diverge (Figs 5 and 7). Although our natural fire scenario is unlikely for WCNP (where prescribed fires now dominate) or for private lands, where fire is typically suppressed, the projected increase in the fire danger indices indicates greater risks of natural wildland fires and future challenges for scheduling prescribed fires.

### Future vegetation

**Figure 8:**
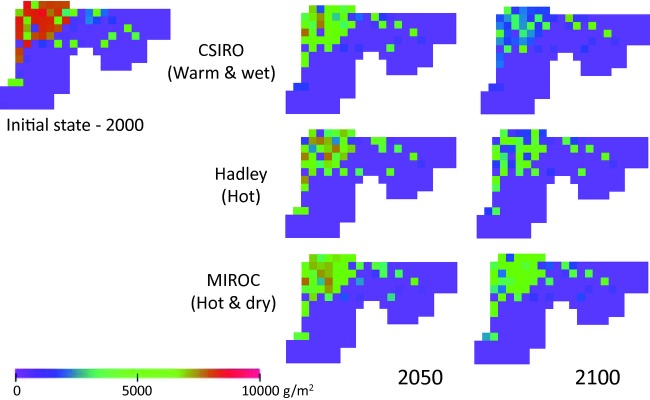
Live tree C with natural fire occurrence simulated by MC1-WCNP using climate projections from three general circulation models; CSIRO Mk3, Hadley CM3 and MIROC 3.2 medres (IPCC 2007), under the A2 emission scenario (Nakićenović et al. 2000). (Initial state based on spinup and historical run with natural fire).

The increase in fire frequency causes a reduction in projected forest biomass by the end of the 21st century, particularly for CSIRO (Fig. 8). No woody invasion of grasslands occurs for any of these scenarios, and grass productivity and biomass decline under the Hadley and especially MIROC climates, particularly during drought years (data not shown). A similar distribution of forests and grasslands is projected when elevated CO_2_ effects in the model are turned off and have no effects on future production or water-use efficiency. But in this case, future plant productivity increasingly diverges below that projected with CO_2_ effects over the 21st century.

**Figure 9:**
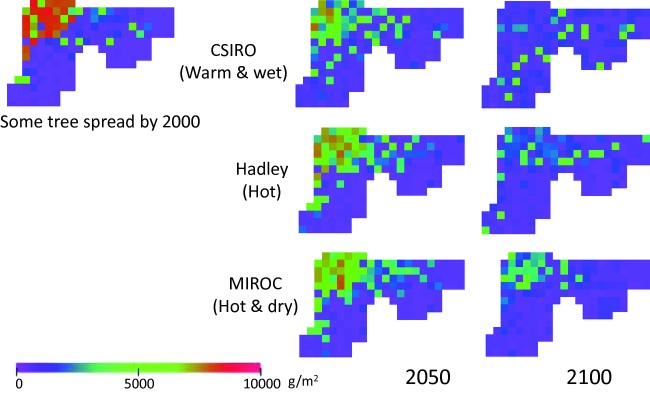
Projected effects of fire suppression starting in 1941 on live tree C simulated by MC1-WCNP with climate projections from three general circulation models; CSIRO Mk3, Hadley CM3 and MIROC 3.2 medres (IPCC 2007), under the A2 emission scenario (Nakićenović et al. 2000). (Simulations based on spinup run with natural fire).

The effects of simulated fire suppression are illustrated in Fig. 9 where suppression is initiated in 1941, the approximate onset of effective fire suppression in the United States (Pyne 1982). A more heterogeneous distribution of forest and woodlands results because small differences in potential fire intensity determine which cells escape fire, yielding a patchwork of burn histories. As grassland fires rarely exceed the suppression threshold, tree incursions into grasslands are projected, depending in part on initial (albeit low) tree biomass.

**Figure 10:**
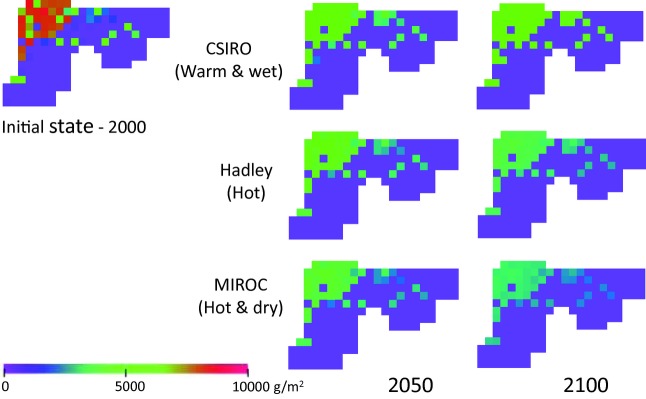
Prescribed fall fire effects on live tree C simulated by MC1-WCNP with climate projections from three general circulation models; CSIRO Mk3, Hadley CM3 and MIROC 3.2 medres (IPCC 2007), under the A2 anthropogenic emission scenario (Nakićenović et al. 2000). All simulated fires were set in mid-September (Table 1). (Initial state based on spinup and historical run with natural fire).

With regularly prescribed fires (and complete suppression of natural fires), projected tree biomass is substantially reduced in cells with high initial biomass, but less so for those with low biomass, resulting in a more homogeneous distribution of projected biomass over the wooded areas (Fig. 10). In this case, cells with low tree biomass recover more quickly than do cells with initially high biomass, where losses due to mortality nearly equal gains due to growth: wooded cells remain wooded and grassland cells remain grassland.

## Discussion

Our application of MC1-WCNP, calibrated to ecotonal vegetation at WCNP, approximates the current proportions of wooded areas and grasslands (Table 2). The simulated distributions of forests and grasslands are largely determined by the simulated fire frequencies across the park. The somewhat hotter and drier conditions in the eastern and southernmost areas of the park result in higher simulated fire frequencies and somewhat lower forest growth rates. In these areas, the simulated tree carbon pools remain small, and the associated low crown base heights result in crown fires that reduce tree biomass to a very low level during the model spinup phase. In contrast, the lower simulated fire frequencies in the northwest portion of the park result in low-mortality surface fires that allow forests to approach their maximum (production-limited) biomass during spinup. Thus, sharp boundaries between forest and grassland are simulated (Fig. 2), depending on whether or not trees grow tall enough to survive fire. These boundaries persist for most future simulations (Figs 10), despite increased fire frequencies, because trees in the northwest part of the park are tall enough to survive simulated fire effects.

However, the model uses an average wind speed, due to a lack of fine-scale wind data, and thus underestimates the range in fire severity. During the warmer 2nd half of the 21st century, it is possible that more frequent fire ignitions could also increase the frequency of fire during times of high wind, thereby generating more crown fires that would affect the resilience of the forest. Observations of recent decreases in the frequencies of high wind speeds for North America (Zhao et al., 2011) and uncertainties in GCM predictions of wind speeds, particularly over land (McInnes et al., 2011), add to the uncertainty in using projected winds to predict future fire regimes and their effects.

### Future fire effects in the Western USA

The projected increase in natural fire frequency at WCNP (Fig. 6) is consistent with other future fire projections for the Western USA. DGVMs generally project an increase in future fire frequency and area burned, although often without decreasing the area occupied by woody vegetation types (Bachelet et al. 2003; Lenihan et al. 2008; Rogers et al. 2011). Increases in future fire effects are also predicted by statistical fire models using trends based on historical climate. For example, Litschert et al. (2012) predicted a 10-fold increase in mean annual burned area from 2011 to 2070 for the Southern Rockies Ecoregion, using the Hadley CM3 A2 climate projection. Similarly, Westerling et al. (2011) predicted large increases in fire frequency over the 21st century for the Greater Yellowstone Ecosystem (GYE), an area characterized by infrequent severe fires with a return interval of 100–300 years. The regionally averaged fire return interval was predicted to decline to 10 years or less by 2080 using future climate projections from 3 GCMs under the A2 emissions scenario. Such a change could threaten the persistence of current conifer species in the GYE (Westerling et al. 2011), but not eliminate ponderosa pine from WCNP because this species is less subject to stand-replacing fires than are the lodgepole pine forests that dominate much of the GYE (Agee 1993).

### Comparison with empirical model predictions for ponderosa pine

Our projection of the persistence of ponderosa pine forests throughout the 21st century differs from those based on empirical relationships between species presence–absence and composite climate variables. Both Shafer et al. (2001) and Rehfeldt et al. (2006) project a contraction in the suitable habitat of ponderosa pine that excludes the Black Hills by the end of the 21st century. Rehfeldt et al. (2006) used random forests multiple-regression tree models, a method that yields accurate assessments of current species–climate associations (Franklin 2009; Wang et al. 2012). They found that for two downscaled and averaged GCM projections,^1^ the climate profile for ponderosa pine would exclude WCNP by 2040 and all of the Black Hills by 2100. This finding justifies concern over the future of ponderosa pine in the park, but its implications for the next century are uncertain because empirical niche-based models do not address the mechanisms nor the time scales over which climate influences species ranges. These models are based on the implicit assumption that the historical distribution of a species is in equilibrium with the historical climate, which may or may not be the case (García-Valdés et al. 2013). Hence, future projections of habitat suitability or species ranges should be viewed as estimates of exposure to climate impacts rather than predictive forecasts (Wiens et al. 2009; Dawson et al. 2011).

In Rehfeldt et al.s' (2006) analysis of ponderosa pine, one of the most important classification variables is the annual moisture index (degree days >5**°**C/mean annual precipitation), which is closely related to the combined gradient of increasing temperature and decreasing precipitation from the northwest to the southeast over WCNP. Over this gradient, a 1**°**C increase in mean temperature and an 8% decline in annual precipitation separate simulated grassland from evergreen needleleaf forest with MC1-WCNP, as calibrated to the observed distribution of ponderosa pine in the park (Fig. 4). However, in reality, this high sensitivity to climate is driven almost entirely by fire effects, as recent observations of pine expansion due to fire suppression have shown (Knight 1999), as have simulations projecting forest over the whole park when fire is turned off (Figs 3 and 4). Fire effects in empirical species distribution models are not explicit, but are implicitly included in the correlation between climate and historical species distribution (Franklin 2009; Vallecillo et al. 2009). Had past fire regimes been different, due to altered human practices, different current species distributions would likely have resulted (Figs 3 and 4), altering the estimates of suitable habitat by these models. Our projection of the persistence of currently wooded areas at WCNP into the future despite substantial warming, due to the fire resistance of mature ponderosa pines, incorporates climate effects on both fire regime and tree growth. In addition, enhanced CO_2_ concentrations increased the simulated water-use efficiency and productivity of both trees and grasses, as compared to the case with CO_2_ effects turned off (fig. 20 in King et al. 2013).

Fire, including that set by indigenous peoples, is a key regulator of the abundance and distribution of many species (Sheuyange et al. 2005; Pausas and Keeley 2009; Bowman and Haberle 2010). Using a process-based DGVM, Bond et al. (2005) projected a doubling of forest cover in a world without fire, and fire effects have been critical in maintaining grasslands across the Great Plains (Anderson 1990; Courtwright 2011). However, fire regimes have been substantially altered over the past century, and fire suppression has allowed a proliferation of fire-sensitive trees over large areas of North America (Agee 1993; Leenhouts 1998; Nowacki and Abrams 2008), including rapid increases in ponderosa pine densities in pine savannas (Knight 1999). Our model projection that ponderosa pine would invade park grasslands without fire is in agreement with current observations of pine seedlings extending into park grasslands up to ∼100 m beyond the nearest trees. Moreover, ponderosa pine seedlings from the Black Hills and Rocky Mountain foothills showed high 15-year survival when planted across the northern, central, and southern Great Plains, including one site (Alliance, Nebraska) that is drier and slightly warmer than areas of the park projected to be grassland by MC1-WCNP (Van Haverbeke 1986). These observations and simulations support the hypothesis that the forest extent in the Black Hills region is driven more by fire regime than direct climate effects.

### Uncertainties

Future fire effects and the influence of extreme events on plant survival and regeneration are major areas of uncertainty in our projections. Our simulation of fire effects is constrained by the interpolation of monthly climate inputs to generate pseudo-daily weather, without including actual fluctuations in temperature, humidity, and wind speed, and by the ignition algorithm, which sets a maximum of one fire per year per cell on the day when ignition thresholds are first exceeded. In reality, spatial heterogeneity in fuel loads and topography and a variety of ignition sources will introduce greater spatial and temporal variability in fire and undoubtedly produce more complex patterns of fire behavior than simulated here (Baker 2009). For example, the greater topographic ruggedness in the currently forested areas of WCNP could contribute to tree survival by limiting fire spread and providing refuges from which trees can reinvade after episodes of greater wildfires (Anderson 1990).

Although adult ponderosa pines are deep-rooted and have high drought tolerance (Niinemets and Valladares 2006), their ability to withstand bark beetle attacks is lessened by drought (Negròn et al. 2009). Bark beetle epidemics, often associated with prolonged droughts, may cause heavy regional mortality of ponderosa pine (Larrson et al. 1983; Bentz et al. 2010). In the Black Hills, the most severe recorded beetle outbreak (1894–1908) killed trees comprising 5–10% of the current wood volume on the Black Hills National Forest (Oliver and Ryker 1990; DeBlander 2002). Intervals of greater drought, as associated with the Hadley and MIROC future climates, would likely increase tree susceptibility to beetle kill (Larrson et al. 1983).

In reality, it is often a combination of interacting factors such as drought and heat stress, pests, pathogens, and competitors that increase tree mortality (Fan et al. 2012). For example, Allen and Breshears (1998) documented a rapid drought-induced shift in the ecotone between ponderosa pine-dominated and pinyon–juniper woodlands in northern New Mexico (USA), a drier region than WCNP. The shift was caused by the death of ponderosa pines along the ecotone during the severe drought of the 1950s, likely exacerbated by competition with coexisting pinyon pines and junipers and a concurrent bark beetle outbreak. Such die-offs of ponderosa pine could occur in other parts of its range if extreme droughts become the norm. Currently, MC1-WCNP, like MC1, simulates drought effects as a reduction in plant production but not as a direct cause of mortality. Mixed modeling approaches (e.g., Keane et al. 2011) are needed to link mortality and other demographic processes to the biogeochemical, biogeographic, and fire disturbance processes included in MC1.

Given these uncertainties, long-term monitoring of tree establishment, growth, mortality, and causes of mortality is essential, particularly along ecotones, where species may be near their range limits (King et al. 2013). This monitoring is crucial for determining the veracity of scientific projections of future vegetation and – more importantly – for making management decisions that do not amplify unwanted vegetation changes driven by climate change.

### General implications

Our projection of climate change effects on the ponderosa pine–grassland ecotone at WCNP illustrates the insights that can be gained from mechanistic models, both in evaluating the drivers of vegetation change and assessing knowledge gaps. For example, other mechanistic models have shown the importance of elevated CO_2_ effects on forest dynamics (Keenan et al. 2011) and the substantial lags in the response of vegetation to changing climates, due in part to tree longevity (Canham 2012; Hickler et al. 2012). Our study suggests that fire can play a major role in mediating climate change impacts on vegetation, as it has in shaping past species distributions (e.g., Bowman and Haberle 2010)

The relative importance of direct climate effects versus other interactions may vary greatly between regions and species. Our results regarding the importance of fire in limiting the historical distribution of ponderosa pine are most applicable to the Black Hills region and possibly the front ranges of the Rocky Mountains where ponderosa pine woodlands abut grasslands. The combination of direct climate effects and pests, pathogens, and fire may limit ponderosa pine in other parts of its range (e.g., Allen and Breshears 1998). Sudden aspen decline observed in trembling aspen (*Populus tremuloides*) stands of western Colorado, USA; and south-central Canada (Worrall et al. 2010; Michaelian et al. 2011) provides an example of dieback due to direct climate effects. Here, the most likely cause of mortality was hydraulic failure of the water-conducting tissues following the intense drought of 2002 (Anderegg et al. 2012). Projected future reductions of suitable habitat for aspen by Rehfeldt et al. (2009) are directly linked to climate, but in this case, fire may also increase shade-intolerant aspen resilience by reducing competition for water and light from later-successional species, such as ponderosa pine (Bartos 2001), as well as promoting natural postfire suckering.

Our results illustrate the importance of disturbance in disequilibrium systems (here exemplified by fire) in determining vegetation patterns and the potential biases in climate change impacts projections that ignore this. For example, niche-based species distribution models, based on the assumption that species are in equilibrium with the recent climate, seldom consider disturbances or the likelihood that their nature and frequency may differ in the future. The inclusion of fire and other disturbances requires ecological as well as statistical expertise to improve the reliability and interpretation of all model projections. A mixture of correlative and process-based modeling approaches involving multiple spatial scales may be most appropriate to address these issues (Keane et al. 2011; Dormann et al. 2012; Kramer et al. 2012).

## Note

1 HadCM3 and CGCM2 with a 1% yr^−1^ increase in greenhouse gases after 1990.

## Appendix 2: Calibration of MC1-WCNP for Wind Cave National Park, South Dakota, USA

### Calibration of MC1-WCNP for ponderosa pine

MC1 has previously been parameterized for large regions that include dense forests with high LAI. In comparison with other trees, ponderosa pine (the dominant tree species at WCNP) is quite drought tolerant and has a low leaf area index (LAI) (Oliver and Ryker 1990; Niinemets and Valladares 2006). We therefore reduced MAXLAI, the LAI asymptote that is approached at very high live wood carbon, from 10 to 5, as supported by the range of LAI values for ponderosa pine forests inferred from the foliage biomass, and LAI values compiled by Cannell (1982). We decreased the sensitivity of tree productivity to water deficits by setting the water effects scalar to 1 when (monthly precipitation + soil water available to trees)/PET >0.8 and ramping it down to 0 as this ratio declines to 0. Following these changes, the maximum net primary production for trees was set at 160 g C m^−2^ mo^−1^. This parameterization yields an LAI of 3–4 and an aboveground live tree C of 7500–9000 g C m^−2^ for old, unburned forests at WCNP, near the upper end of the range inferred from plot measurements by park personnel (A. Symstad & D. Swanson, unpubl. data).

Before the advent of fire suppression, ponderosa pine forests were subject to less severe fire effects than those typically simulated by MC1 (Allen et al. 2002), and surface fires were common in the Black Hills in presettlement times (Brown and Sieg 1999). Hence, we altered the fire module such that simulated crown fires are rare in the more open mature forests that developed under presettlement fire frequencies in this area. This was accomplished by defining the flashy 1-h fuels as an adjustable fraction of the surface litter pool (calibrated at 86%) plus all standing dead grass and drought-cured live grass. Because ponderosa pine is more fire resistant than most other trees, we replaced the previously used general mortality function of Ryan and Reinhardt (1988) with that of Hood (2008) for ponderosa and Jeffrey pine, yielding:

(A1)

where ck is the fraction of the crown killed by fire. We removed a mortality threshold in MC1 above which mortality is set to 100%, thereby simulating more burns with partial mortality. We also altered the allometric relationship between tree height and biomass (used to compute crown fire occurrence, which depends on crown base height) based on data for ponderosa pine in the Black Hills (Appendix 3; King et al. 2013; Tree Allometry section).

MC1 includes a minimum fire effect that is user-assigned. The rationale for minimum mortalities is that the model calculates fire effects assuming homogeneous fuel and wind conditions across each cell and does not account for hot spots in an otherwise sublethal fire. For WCNP, we used an adjustable minimum crown kill fraction, which is translated into mortality by equation (A1). This method better links whole tree mortality and crown kill. We set the minimum crown kill fraction at 0.10, which yields 6.26% mortality, based in part on our objective of simulating the observed proportions of wooded areas in the park.

MC1 simulates partial burn in a grid cell when the time since the last fire is less than the vegetation type-dependent threshold. The fraction of the cell burned = (years since last fire)/(fire return threshold). The calculated mortality and other simulated effects are then multiplied by this fraction, thereby reducing fire effects. Partial burn was designed to limit fire effects when the scale at which the model was run was large (cell size of ∼50 × 50 km).

Based on presettlement fire histories for the southern Black Hills (Leenhouts 1998; Brown and Sieg 1999), we set the fire return threshold to 10 years for the temperate evergreen needleaf forest and woodland and the temperate and subtropical shrubland vegetation types and retained the 7-year threshold for grassland vegetation types. The 10-year return interval for wooded areas is approximately the time required for the fine fuels to increase to prefire levels. Given the heterogeneity in fuels not included in our model, it is increasingly likely that actual fires will skip areas as burn frequency increases.

We also altered the allometric algorithm used to estimate tree dimensions from the live tree carbon pools, as required to calculate fire effects. We related tree height to live aboveground woody biomass per unit ground area based on data for pines of North America from the worldwide forest biomass compendium of Cannell (1982) and used data of Symstad and Bynum (2005) to relate trunk diameter to height for ponderosa pine of the Black Hills.

### Additional model adjustments

Competition between grasses and trees is ensured in MC1 using the original savanna mode of the CENTURY model, which calculates both grass and tree NPP as affected by soil temperature, water availability, and shading of grass by trees. Tree production in MC1, as in CENTURY, is multiplied by a scalar that increases from a value of 0 for an LAI of 0 toward an asymptote of 1 at high LAI values. For this calculation, tree LAI is capped at a minimum value of 0.2 to prevent the elimination of the woody life-form in savanna mode. This approach is also reasonable for simulating regeneration following crown fires that are assumed to kill all trees – as regeneration from seeds or root sprouts may soon establish a new tree canopy under favorable conditions. However, this approach also simulates vigorous tree establishment in fire-maintained grasslands that are far from the nearest tree. We therefore greatly reduced the minimum value of LAI that is used in calculating this scalar to a value of 0.000001. To enable the regeneration of trees following crown fires, we lowered the crown fire mortality rate from 100% to 98%, which ensures adequate postfire tree NPP to regenerate burned forests, unless the fire frequency is high. These changes produce a much sharper woodland–grassland boundary, as is commonly observed in the Black Hills region.

Based on observed values of standing grass biomass at WCNP by park personnel, we set the maximum grass production rates at 150 and 200 g C m^−2^ mo^−1^ for C_3_ and C_4_ grasses, respectively. Based on reviews of grazing impacts on rangelands (Milchunas and Lauenroth 1993; Holecheck et al. 1999), we assumed no grazing impact on grass production for removal rates of 0 to 30% and a quadratic decline to zero production for removal rates of 30–90%.

MC1 calculates the direct effects of atmospheric CO_2_ concentrations on both production and transpiration as logarithmic functions of (current CO_2_ concentration)/350 ppm. When this ratio is 2 (i.e., ambient CO_2_ = 700 ppm), production is multiplied by a factor of 1.25 for both trees and grasses, and transpiration is multiplied by a factor of 0.75, thereby reducing projected water deficits for a given canopy cover. However, recent long-term open-air CO_2_ enrichment studies suggest lower and variable CO_2_ impacts on production, but consistent increases in water-use efficiency (Körner et al. 2005; Nösberger et al. 2006; Norby et al. 2010; Lee et al. 2011; Morgan et al. 2011; Warren et al. 2011). Thus, we have retained the above parameterization for transpiration, but have reduced the production multiplier for doubled CO_2_ from 1.25 to 1.15 for both trees and grasses.

**Table A2:** Parameter changes for MC-WCNP. Values from Bachelet et al. (2001) for MC1 are shown in parentheses.

Parameter file	Parameter	Common value	C3	C4
crop.100	PRDX(1)		150 (300)	200 (400)
crop.100	CO2IPR	1.15 (1.25)		

Parameter file	Parameter	Common value	Dec. Needleleaf	Ev. Needleleaf	Dec. Broadleaf	Ev. Broadleaf
tree.100	PRDX(4)		250	160 (250)	160 (250)	250
tree.100	MAXLAI		5 (10)	5 (10)	5 (10)	5 (10)
tree.100	CO2IPR	1.15 (1.25)				
Parameters	evergreen_selection1	4 (12)				

## Appendix 3: Changes to code described in Appendix 2. Code file name listed in bold print. Listed are extracts from much larger files

fuel_load.c

/* Ponderosa allometry follows, superceding previous allometry DK */

if (ltree2 < 4000.0) tree_ht = 0.4689 * pow(ltree2,(1./3.));

else tree_ht = 0.04071 * pow(ltree2,0.628);

if (tree_ht > 27.0) tree_ht = 27.0;

if (tree_ht < 4.052) dbh = 1.3 * tree_ht;

else dbh = 0.891 * pow(tree_ht,1.27);

/* Changed fuel partitioning follows - DK */

d_1 hr = 0.86 * littr + dstnd;

d_10 hr = 0.5 * dwod1;

d_100 hr = 0.5 * dwod1;

d_1000 hr = dwod100;

fire_sched.c

cen_state[2] = 0.; /* Correction so that fire-killed trunks are not considered to be consumed. DK */

/* changes to cen_state vars below (which are passed to the biogeochemistry module) - done in concert with fuel load changes DK */

cen_state[3] = (0.5 * fireVars.fire_eff.consume[burn_day][4]

+ 0.5 * fireVars.fire_eff.consume[burn_day][5]) * part;

cen_state[4] = fireVars.fire_eff.consume[burn_day][6] * part;

cen_state[7] = (0.86 * fireVars.fire_eff.consume[burn_day][3]

+ 0. * fireVars.fire_eff.consume[burn_day][4]

+ 0. * fireVars.fire_eff.consume[burn_day][5]

+ 0. * fireVars.fire_eff.consume[burn_day][6]) * part;

c The following changes affect the calculation of partial burns.

case 4: /* BOREAL EVERG FOR */

min_mfri = 10.; /* Changed from 50 DK Oct 4, 012 */

max_mfri = 10.;

break;

case 5: /* BOREAL MIXED FOR */

min_mfri = 10.; /* Changed from 50 DK Oct 4, 012 */

max_mfri = 10.;

break;

case 8: /* TEMP EVERG FOR */ /* Changed from 50. -DK */

min_mfri = 10.;

max_mfri = 10.;

break;

case 9: /* TEMP DECID FOR */

min_mfri = 10.; /* Changed from 100 DK Oct 4, 012 */

max_mfri = 10.;

break;

case 10: /* TEMP COOL MXD FOR */

min_mfri = 10.; /* Changed from 100 DK Oct 4, 012 */

max_mfri = 10.;

break;

case 12: /* TEMP EVERG WOODL */ /* Changed from 15. -DK */

min_mfri = 10.;

max_mfri = 10.;

break;

case 16: /* TEMP SHRUBL */ /* Changed from 30. -DK */

min_mfri = 10.;

max_mfri = 10.;

break;

case 37: // coniferous xeromorphic woodland

// from case 27: /* SUBTROP SHRUBL */ /* Changed from 30. DK */

assert(biogeog_option==WWETAC_BIOGEOG);

min_mfri = 10.;

max_mfri = 10.;

break;

fire_eff.c

if (ck < 0.)

ck = 0.0;

if(ck < fire_min) ck = fire_min; /* fire_min applied to crown kill fraction rather than directly to mortality function DK */

if (ck > 1.0)

ck = 1.0;

/* For ponderosa and jeffry pine - DK 2011 */

prob_mort = 100. * (1. / (1. + exp(2.7103 - 4.093 * pow(ck, 3.))));

if (crown_fire)

{

consumed[1] = 98.; /* consumed & killed changed from 100% to lower value here and below DK */

consumed[2] = 98.;

killed[0] = 0.;

killed[1] = 0.;

killed[2] = 98.;

killed[3] = 98.;

}

potfor.F

if (.not. pprdwc_skip_code_flag) then

if (pprdwc_old_code_flag) then

mode = 1

pptprd = pprdwc_old(wc,pptprd,pprpts,mode)

else

c Change to water limitation scalar below makes trees less sensitive to water deficits. DK

pptprd = pprdwc(wc, pptprd, 0., 0., 0.8)

endif

endif

laprod.F

c Large reduction in minimum value for lait (tree LAI) used in this function only (i.e. tree LAI is unchanged outside this function) - DK

if (lait .lt. 0.000001) then

lait = 0.000001

endif

laprod = 1.0 - exp(laitop * lait)

sumcar.F

c Reduced lower limits on the following by a factor of 10 (1st 2) or 100 (last 3), thereby allowing lait to approach minimum value in laprod.F -DK

if (rlvcis(1) .lt. 0.0001) rlvcis(1) = 0.0001

if (frtcis(1) .lt. 0.0001) frtcis(1) = 0.0001

if (fbrcis(1) .lt. 0.0001) fbrcis(1) = 0.0001

if (rlwcis(1) .lt. 0.001) rlwcis(1) = 0.001

if (crtcis(1) .lt. 0.001) crtcis(1) = 0.001
